# Acute and Prolonged Effects of Sweeteners and Sweetness Enhancers on Postprandial Substrate Oxidation, Energy Expenditure, Glucose, and Insulin in Humans—A SWEET Sub-Study

**DOI:** 10.3390/nu18172863

**Published:** 2026-09-02

**Authors:** Sabina S. H. Andersen, Louise Kjølbæk, Jason C. G. Halford, Joanne A. Harrold, Anne Raben

**Affiliations:** 1 Department of Nutrition, Exercise and Sports, Faculty of Science, University of Copenhagen, 1958 Frederiksberg, Denmark; sabina@nexs.ku.dk (S.S.H.A.); louisekjoelbaek@nexs.ku.dk (L.K.); 2 Department of Psychology, University of Leeds, Leeds LS2 9JT, UK; j.halford@leeds.ac.uk; 3 Department of Psychology, University of Liverpool, Liverpool L69 7ZX, UK; harrold@liverpool.ac.uk; 4 Clinical Prevention Research, Copenhagen University Hospital–Steno Diabetes Center Copenhagen, 2730 Herlev, Denmark

**Keywords:** energy balance, body weight regulation, obesity, glucose regulation

## Abstract

**Background/Objectives:** Sweeteners and sweetness enhancers (S&SEs) have been proposed to impair energy metabolism acutely and over time. **Methods:** This sub-study investigated the acute effects of a mixture of acesulfame potassium and cyclamate (Ace-K/Cyc) versus water on postprandial substrate oxidation, energy expenditure, and glucose and insulin concentrations at baseline, after a 2-month weight loss period, and after a 4-month weight loss maintenance period including or excluding S&SEs in the diet. In both groups, allowed added sugar intake was <10 energy-%. A total of 26 participants (18–65 years; BMI ≥ 25.0 kg/m^2^) were recruited from the 1-year randomized controlled SWEET trial, and 16 completed the final test day. Fasting measurements were taken (ventilated hood; blood samples) before a standardized breakfast. Hereafter, postprandial measurements were taken until time point 250 min. At time point 120 min, a test drink containing either Ace-K/Cyc or water was served. **Result:** There were no differences between the two groups in fasting or postprandial fat oxidation (primary outcome), carbohydrate oxidation, energy expenditure, glucose, or insulin (secondary outcomes) on or across the three test days (*p* > 0.05). **Conclusions:** There were no statistically significant differences between Ace-K/Cyc and water in postprandial fat oxidation, carbohydrate oxidation, energy expenditure, or glucose or insulin either acutely, after a weight loss period, or after a 4-month period including or excluding S&SEs in the diet. The low number of participants should, however, be considered a limitation of our conclusions.

## 1. Introduction

Non-caloric sweeteners (NCS) are food additives that possess high-intensity sweetening properties while containing little or no energy [1,2]. Low-caloric sweeteners (LCS) are food additives that provide sweetness along with a reduced energy content (typically around 7.7 kJ/g) compared to, e.g., sucrose (16.8 kJ/g) [1,3,4]. As a group, NCS and LCS can be defined as sweeteners and sweetness enhancers (S&SEs). Sweeteners and sweetness enhancers can preserve food palatability without providing the associated energy or glycemic impact [1,3,5,6,7]; however, their inertness is a highly debated matter [2,8,9,10,11,12,13,14,15].

The debate reached a turning point with the recently published recommendation by the World Health Organization (WHO) [16]. Drawing upon a systematic review from 2022 [17], the WHO advises against using NCS for, e.g., weight management, arguing that the systematic review showed no long-term benefits in reducing body fat while suggesting increased risk of, e.g., non-communicable diseases associated with consuming NCS [16,17]. Contrasting with the WHO recommendation are a 2020 expert panel’s consensus statements concluding, e.g., that NCS promote weight loss, do not negatively affect glucose regulation, and should be considered as a strategy to reduce sugar intake [18]. The discrepancy between the two recommendations most likely relates to how the current evidence is weighted; in the WHO recommendation, randomized controlled trials (RCTs) and observational studies seem to be weighted equally, while RCTs are weighted higher than observational studies in the expert panel’s consensus statements [16,18]. Observational studies are at risk of confounding and reverse causality, and, for the same reason, the recommendation by the WHO is conditional [16,19]. The contrast between the two recommendations clearly highlights the differences between the results of RCTs and observational studies within this research area.

Sweeteners and sweetness enhancers have been proposed to potentially contribute to weight gain by stimulating sweet taste receptors beyond the perception of sweetness, similar to caloric sweeteners [8,9,10,12,14,15], and/or by disturbing the gut microbiome [11,13]. This could lead to impaired sensory and endocrine signaling [8,9,10,11,12,13,14,15]. Previous reviews, however, find no overall negative effects of NCS and/or LCS on appetite and glucose regulation [3,7,20,21,22,23,24]. The role of S&SEs on the other side of energy balance, i.e., substrate oxidation and energy expenditure, was evaluated in a recently published systematic review by Andersen et al. [25]. The systematic review found that drinks and meals with NCS or LCS resulted in higher fat oxidation and lower carbohydrate oxidation compared to caloric sweeteners (e.g., sucrose) under non-isoenergetic conditions. This finding was attributed to the extra carbohydrate load in the interventions with caloric sweeteners [25]. Thus, comparing S&SEs to caloric sweeteners under non-isoenergetic conditions may mask any potential effect of S&SEs. The results for energy expenditure were inconsistent, and no other conclusions could be made due to insufficient and inconsistent data [25]. The systematic review showed a need for further studies using isoenergetic comparators (e.g., water) and of longer duration [25].

Mainly, NCS seem to be the target of the sweet taste receptor theory [8,9,10,12,14,15]. If NCS can activate sweet taste receptors beyond the perception of sweetness, they might temporarily suppress fat oxidation and increase carbohydrate oxidation and energy expenditure, thus resembling the effect of carbohydrates [25]. Looking at the long-term effects, both gut dysbiosis caused by S&SEs and repeated activation of cephalic-phase responses by NCS without the associated energy load have been proposed to impair carbohydrate metabolism [9,10,11,12,13,15]. Both of these long-term effects could potentially result in inadequate inhibition of fat oxidation and insufficient elevation of carbohydrate oxidation and energy expenditure postprandially [25].

The relevance of the above mechanisms for specific NCS or LCS depends on their individual metabolism [2,7]. Human data looking at the NCS acesulfame potassium (Ace-K) and cyclamate (Cyc) in relation to the outcomes of interest are scarce [25]. Neither Ace-K nor Cyc are broken down into by-products in the intestine, as, e.g., aspartame [1,2]. Thus, they have the potential to affect sweet taste receptors not only in the oral cavity but also in various tissues such as the brain, adipose tissue, and pancreas [2,26,27]. Specifically, Ace-K has been shown to stimulate adipogenesis and inhibit lipolysis in vitro [9,28].

The aim of this sub-study was therefore to investigate the effects of a mixture of Ace-K and Cyc on postprandial fat oxidation (primary outcome), carbohydrate oxidation, energy expenditure, and glucose and insulin concentrations (secondary outcomes) compared to water. This investigation was carried out before the intervention (baseline), after a 2-month weight loss (WL) period, and after a 4-month weight loss maintenance (WLM) period including or excluding S&SEs in the diet. We hypothesized that there would be no difference in postprandial fat oxidation between the two intervention groups at baseline, after WL, or after WLM with S&SEs in the diet.

## 2. Materials and Methods

### 2.1. Study Design and Participants

The sub-study was conducted between September 2020 and March 2022 at the Department of Nutrition, Exercise, and Sports, University of Copenhagen, Denmark. Participants were recruited from a 1-year RCT as part of the European Horizon 2020 project SWEET (‘Sweeteners and Sweetness Enhancers: Impact on Health, Obesity, Safety and Sustainability’) (http://www.sweetproject.eu (accessed on 1 August 2026)) [29]. The aim of the RCT was to investigate whether prolonged consumption of S&SEs would improve 1-year WLM as well as obesity-related risk and safety markers compared to excluding S&SEs, both within a healthy diet regime [29]. The main study, including the sub-study, was registered at www.clinicaltrials.gov (accessed on 1 August 2026) (NCT04226911), approved by the research ethics committees of the capital region (Denmark) (H-19040679), and conducted in accordance with the Declaration of Helsinki. All study participants signed informed consent forms before participating in any study-related activities.

**Figure 1:**
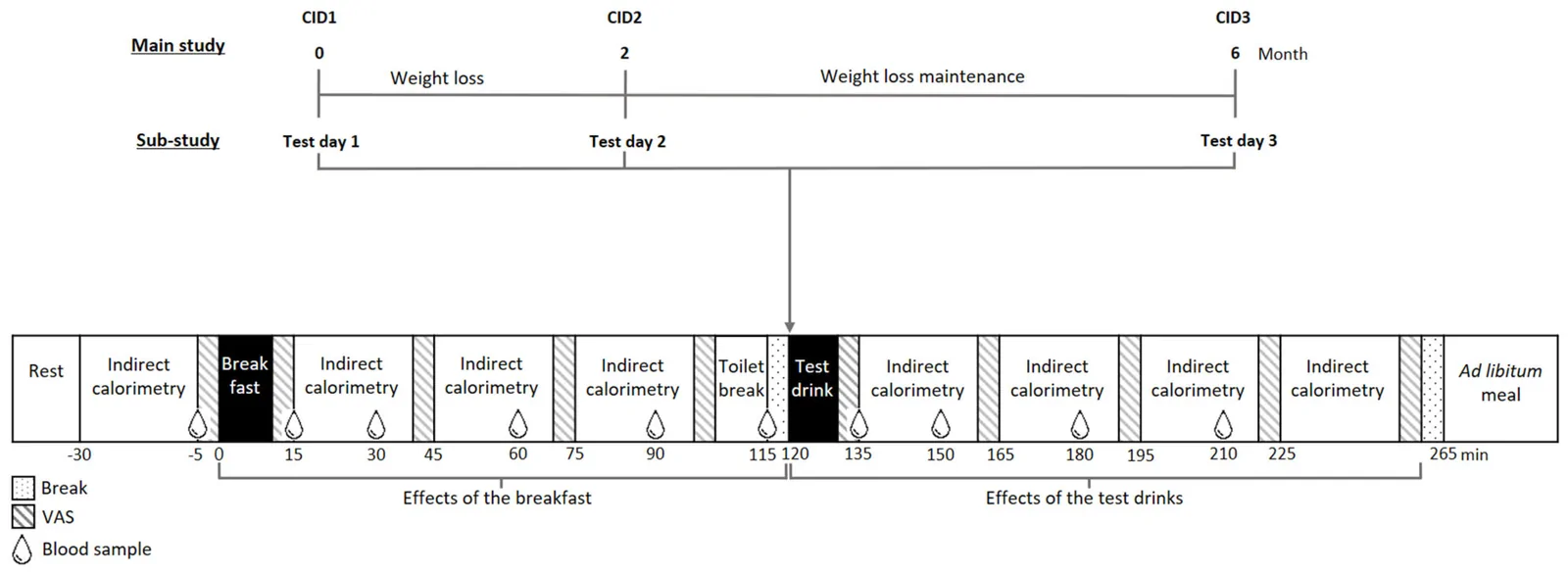
Study design of the main study and sub-study including a detailed layout of the study procedures performed on each test day. CID4 is not included in the illustration of the main study. Abbreviations: CID, clinical investigation day; VAS, visual analogue scales.

The design of the main study has previously been published [29], as have the main results (Pang et al. 2025) [30]. Both adults and families who were overweight/obese were recruited, but information (intervention, outcomes, etc.) about children will not be covered here, as only adults participated in the sub-study. Inclusion criteria were age 18–65 y, body mass index (BMI) ≥ 25.0 kg/m^2^, and regular consumption of sugar-containing products. Exclusion criteria were, e.g., chronic diseases (e.g., diabetes, coeliac disease) or medication that could interfere with the study outcomes [29]. The main study consisted of 4 clinical investigation days (CIDs) scheduled before WL (month 0), after WL (month 2), during WLM (month 6), and after WLM (month 12) (Figure 1). During the WL period, all adult participants consumed a low-energy diet (LED) with the aim of reducing body weight by at least 5%. The LED consisted of powered shakes, soups, smoothies, and bars from Cambridge Weight Plan (Northants, UK). Participants were allowed to consume up to 4 products pr. day providing 3347–4186 kJ/day [29]. After the WL period, participants were allocated into 2 different intervention groups (the S&SEs group or the Sugar group) which either included or excluded foods and drinks containing S&SEs [29]. Randomization was performed after screening with a 1:1 ratio in blocks of 4 stratified by sex, age, and BMI using a randomization list created by a person not involved in the RCT [29]. The randomization was not revealed to the participants before the completion of the WL period [29].

For the sub-study, the aim was to include a total of 48 Danish participants from the main study with an equal distribution of men, women, and participants from each intervention group. The sub-study included 3 additional test days: test day 1, month 0 (baseline), scheduled 7–10 days before CID1; test day 2, scheduled 7–10 days after CID2; test day 3, scheduled 7 days before or after CID3 (Figure 1). On test day 1, the acute effects of a standardized breakfast and a test drink with either Ace-K/Cyc or water were investigated. On test day 2, the same was investigated, but, on this test day, participants had lost >5% of their body weight. On test day 3, it was investigated whether a 4-month period including or excluding S&SEs affected the acute response to a standardized breakfast and a test drink with either Ace-K/Cyc or water. Participants, but not the investigators, were blinded in the sub-study.

Prior to the 3 test days, participants were instructed to fast for a minimum of 10 h, and to not perform high-intensity physical activity, drink coffee, or smoke for 12 h. A total of 0.5 L of water was allowed. Each test day lasted approximately 6 h. On each test day, participants arrived at 8 o’clock. Fasting body weight was measured, and, in a sub-group (n = 15), a catheter for blood sampling was inserted into the elbow joint. After 15 min bed rest, fasting substrate oxidation and energy expenditure were measured via indirect calorimetry, and appetite sensations and blood samples were taken. A standardized breakfast was served and the measurements repeated until 120 min. Then, the test drink was served and the measurements repeated until 240 min, after which an ad libitum meal was served. Results for appetite sensations and ad libitum energy intake have been published separately (Andersen et al. 2026) [31]. During the test days, a planned toilet break was allowed at 105 min (Figure 1).

Indirect calorimetry was measured through a ventilated hood system (Jaeger Oxycon PRO, Viasys Healtcare GmbH, Höchberg, Germany) and substrate oxidation and energy expenditure were calculated using the Elia and Livesey method [32] assuming constant protein oxidation (1741 kJ/day). Substrate oxidation and energy expenditure were measured 8 times per test day, with each measurement lasting 25 min (Figure 1). The first 5 min of each measurement were not included in the calculations. Thus, the average substrate oxidation and energy expenditure measured from, e.g., time point 15 to 40 min, was calculated from the interval 20–40 min (reported in the figures as time point 30 min). Ambient conditions and gas calibration were checked between all measurements. Participants were allowed to listen to podcasts or music, but were instructed not to talk or sleep during the measurements.

Blood samples were drawn 10 times per test day through a peripheral venous catheter (Figure 1). Glucose and insulin levels were analyzed using a Pentra 400 analyzer (HORIBA ABX, Montpellier, France) and an Immulite 2000xpi analyzer (Siemens Healthcare Diagnostics product INC, Flanders, NJ, USA), respectively.

Total postprandial outcomes were calculated via net incremental area over/under the curve (netAOC/AUC) using the trapezoidal rule. The netAOC/AUC was divided into 3 time periods, thus representing the effects of the breakfast, the test drink, and the total time period (Figure 1). For substrate oxidation and energy expenditure, these were 0–90 min, 90–240 min, and 0–240 min. For glucose and insulin, these were 0–115 min, 115–210 min, and 0–210 min. For substrate oxidation and energy expenditure, the 90 min time point was used as the fasting measurement when calculating the 90–240 min netAOC/AUC. For insulin and glucose, the 115 min time point was used as the fasting measurement when calculating the 115–210-min netAUC.

### 2.2. Test Meals

The standardized breakfast included Arla Cultura^®^ (sour yogurt) with oats and cranberries served with 250 g water (Table 1). Participants randomized to the S&SEs intervention received a test drink containing 5 g of Atwell^®^-0-calories^®^ (sweetness intensity equal to 50 g sugar) and 400 g of water. Atwell^®^ is a fluid product and contains a mixture of Ace-K (3.2%) and Cyc (3.2%). Participants randomized to the Sugar intervention received 405 g of pure water (Table 1). To align with the nomenclature from our previous publications in the SWEET trial [29,30,31], we have kept the name “Sugar group” for the group who received water as a test drink on the 3 test days. For both the breakfast and test drinks, participants were instructed to consume the entire meal/drink over 10 min, meaning that the last bite/sip should be consumed in the 10th minute. No talking was allowed while consuming the meal/drink.

### 2.3. Statistical Power

A clinically meaningful difference in fat oxidation was considered to be 0.017 g/min, as this would amount to a difference between groups of 750 g fat oxidation, or, theoretically, 1 kg body WL per month. With a standard deviation (SD) of ±0.019 g/min, 80% power, a significance level of 0.05, and an expected dropout rate of 20%, 48 participants should be recruited (n = 24) in each intervention group.

### 2.4. Statistical Analysis

Statistical analyses were performed in R version 4.2.1 (R Core Team, 2022, Vienna, Austria). Initial data cleaning involved visual inspection of individual curves, and no outliers (data points outside ±3 SD) were found. All outcomes were analyzed both as repeated measurements and netAOC/AUC via mixed linear ANCOVA models. Models for repeated measurements included interactions between test day, time, and meal, and were adjusted for participant number, test day, and hood machine used (when relevant) as random effects, and age, sex, smoking status, BMI at baseline, weight change from baseline, and fasting value (0 min) of the specific outcome as fixed covariates. In case of a time–meal–test day, time–meal interaction, or meal effect, time points/estimated mean differences were compared using emmeans (estimated marginal means) in R. Models for netAOC/AUC included interactions between meal, period, and test day, and were adjusted for participant number, test day, and hood machine used (when relevant) as random effects, and age, sex, smoking status, BMI at baseline, and weight change from baseline as fixed covariates. Estimated mean differences were again calculated using the R function emmeans. Holm’s method was used for multiple testing adjustments when differences were found. All models were run without weight change from baseline to ensure that any effect caused by the weight loss combined with Ace-K/Cyc was not masked. This did not change the results; thus, only results including this covariant are shown. Model validation was checked by normal quantile and residual plots. After log transformation of the model for repeated measurements of insulin, all models were deemed valid. Available-case analyses were chosen due to high dropout rates. Two participants received the wrong test drink on test day 1 but received the correct test drink going forward. Since they were included in the analyses according to the test drink received on the given test day, this mix-up does not alter the results of our analysis. When calculating baseline characteristics, the 2 participants were included in the group they originally belonged to. Baseline characteristics are shown as means ± SD, while all figures illustrate unadjusted means ± standard error mean (SEM). Significance level was *p* < 0.05. The adjusted between-group difference and 95% confidence intervals are shown in Supplemental Table S1.

## 3. Results

### 3.1. Study Flow

**Figure 2:**
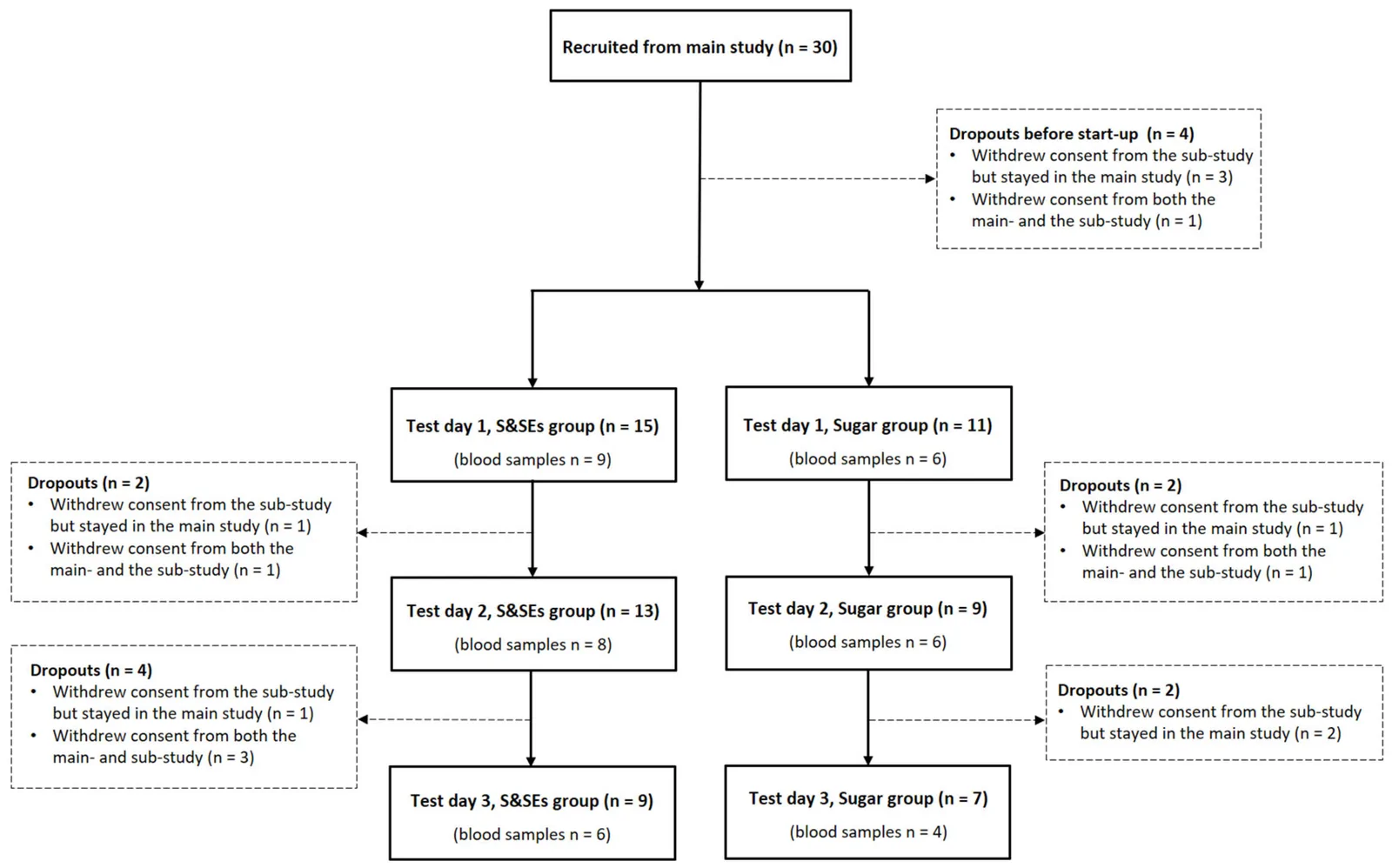
Flow chart showing the flow of participants throughout the sub-study. Abbreviations: S&SEs, sweeteners and sweetness enhancers.

Thirty participants were recruited for the sub-study—26 completed test day 1, 22 test day 2, and 16 test day 3. Reasons for dropping out were, e.g., demanding study activities, participants feeling uncomfortable in the ventilated hood, or personal reasons (Figure 2).

### 3.2. Baseline Characteristics

Baseline characteristics did not differ between the two groups (Table 2), or between completers and dropouts (all *p* > 0.05). No differences were found for fasting fat, carbohydrate oxidation, energy expenditure, glucose, or insulin on the three test days, or when comparing changes in fasting measurements between the three test days (all *p* > 0.05) (Table S1).

### 3.3. Fat Oxidation

**Figure 3:**
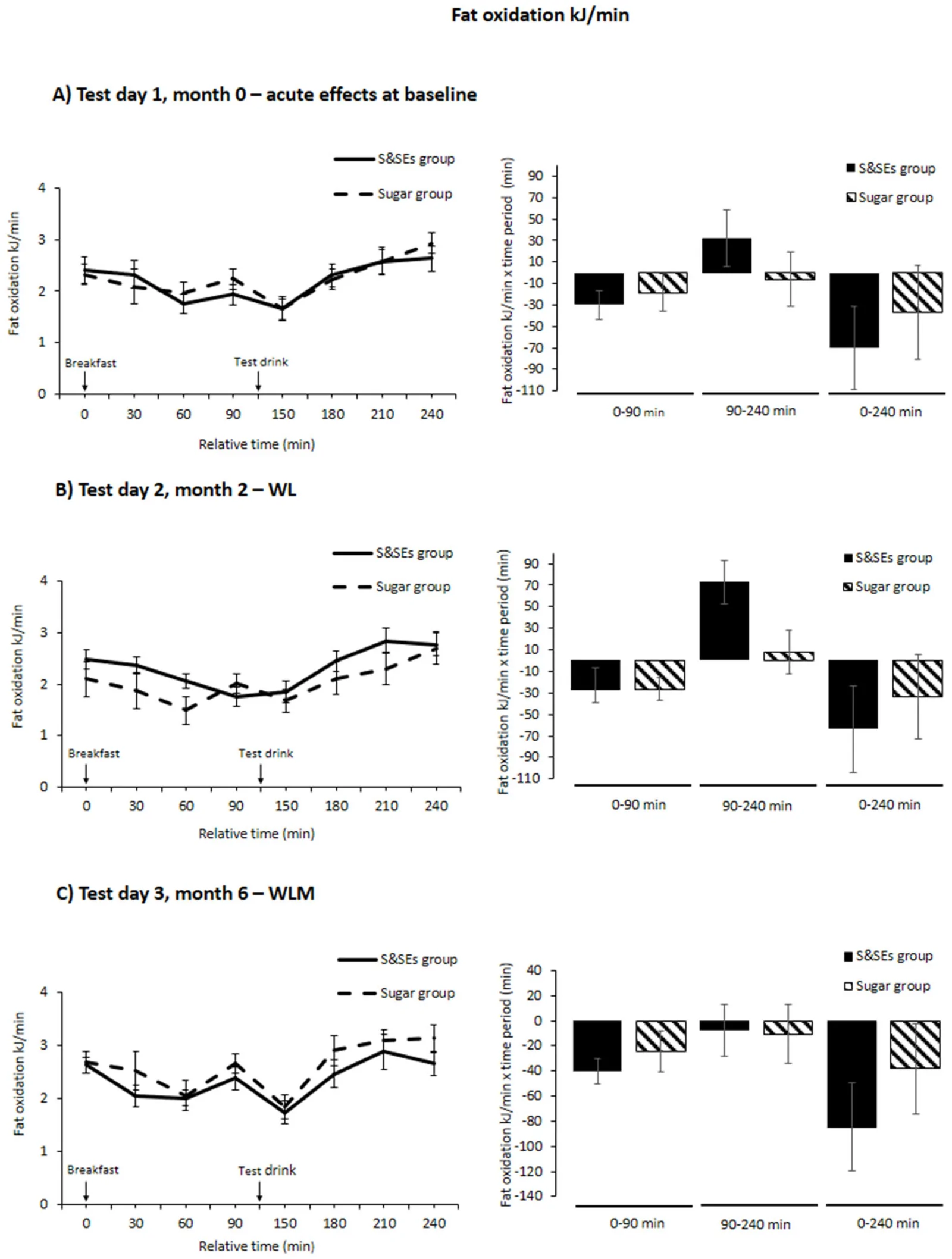
Fat oxidation as unadjusted means ± SEM at different time points (**left**) and netAOC (**right**) for all 3 test days: (**A**) test day 1, month—acute effects at baseline (S&SEs group, n = 14; Sugar group, n = 11, data from 1 participant were lost due to technical issues), (**B**) test day 2, month 2—WL (S&SEs group, n = 13; Sugar group, n = 9), and (**C**) test day 3, month 6—WLM (S&SEs group, n = 9; Sugar group, n = 7). To illustrate that netAOC provides information about how much fat oxidation is inhibited, the netAOC means have been reversed. ANCOVA models were used to test for differences between groups. The models all included participant number, test day, and hood machine as random effects, and age, sex, smoking status, BMI at baseline, and weight change from baseline as fixed effects. Models for repeated measurements also included fasting fat oxidation as a fixed effect. No differences were found between groups (all *p* > 0.05, adjusted means). Abbreviations: netAOC, net area over the curve; SEM, standard error mean; S&SEs, sweeteners and sweetness enhancers; WL, weight loss; WLM, weight loss maintenance.

There was no time–meal–test day interaction (*p* = 0.65), time–meal interaction (*p* = 0.11), or meal effect (*p* = 0.23) for fat oxidation (Figure 3, left). Furthermore, the netAOC for fat oxidation did not differ between groups for any of the time periods (0–90 min, 90–240 min, 0–240 min) on any test day (Figure 3, right; Supplementary Table S2), or when comparing changes in netAOC between the three test days (all *p* > 0.05).

### 3.4. Carbohydrate Oxidation

**Figure 4:**
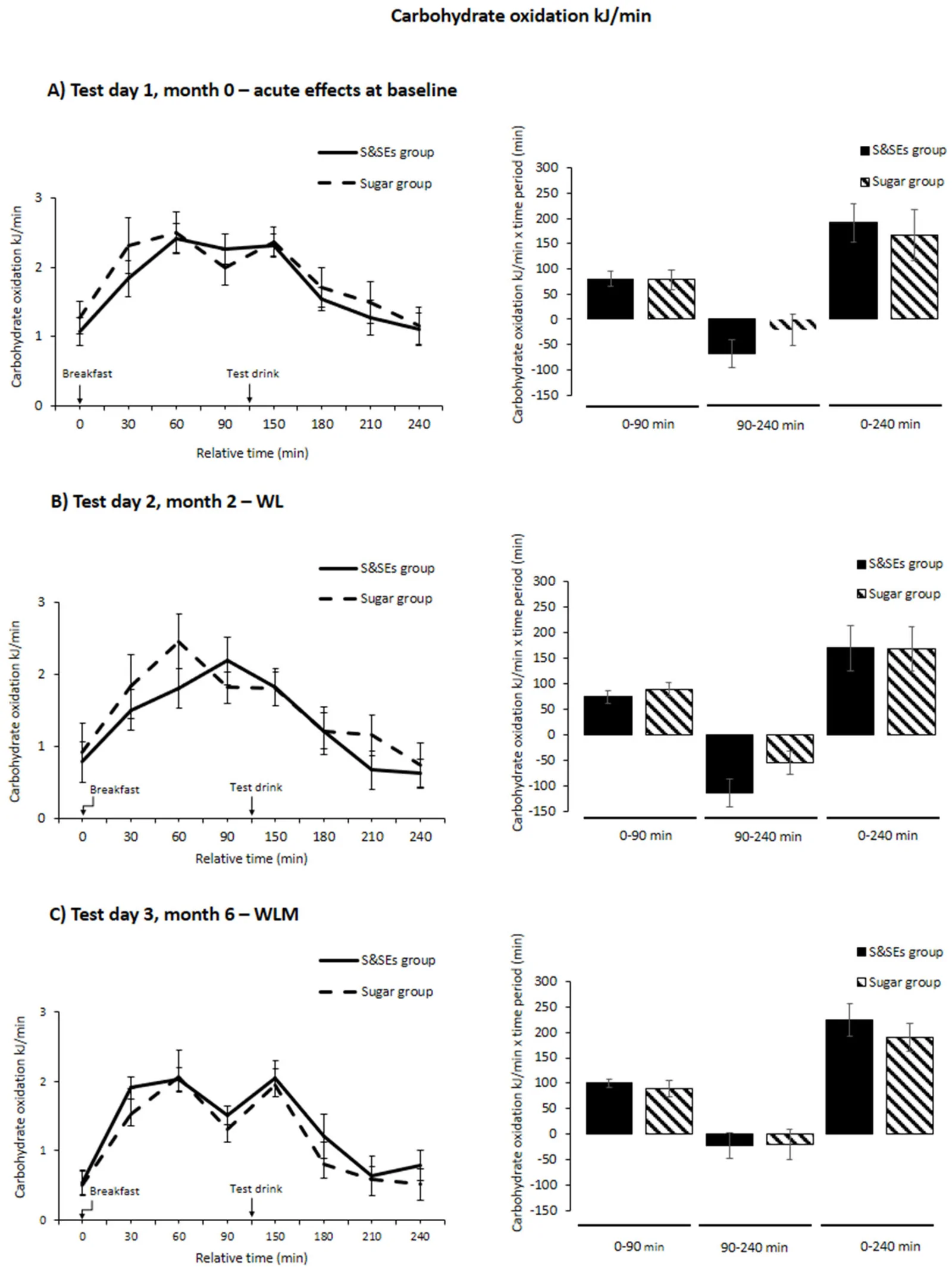
Carbohydrate oxidation as unadjusted means ± SEM at different time points (**left**) and netAUC (**right**) for all 3 test days: (**A**) test day 1, month 0—acute effects at baseline (S&SEs group, n = 14; Sugar group, n = 11, data from 1 participant was lost due to technical issues, (**B**) test day 2, month 2—WL (S&SEs group, n = 13; Sugar group, n = 9), and (**C**) test day 3, month 6—WLM (S&SEs group, n = 9; Sugar group, n = 7). ANCOVA models were used to test for differences between groups. The models all included participant number, test day, and hood machine as random effects, and age, sex, smoking status, BMI at baseline, and weight change from baseline as fixed effects. Models for repeated measurements also included fasting carbohydrate oxidation as a fixed effect. No differences were found between groups (all *p* > 0.05, adjusted means). Abbreviations: netAUC, net area under the curve; SEM, standard error mean; S&SEs, sweeteners and sweetness enhancers; WL, weight loss; WLM, weight loss maintenance.

There was no time–meal–test day interaction (*p* = 0.70), time–meal interaction (*p* = 0.11), or meal effect (*p* = 0.95) for carbohydrate oxidation (Figure 4, left). Furthermore, netAUC carbohydrate oxidation did not differ between groups for any of the performed analyses (Figure 4, right; Supplementary Table S2) (all *p* > 0.05).

### 3.5. Energy Expenditure

**Figure 5:**
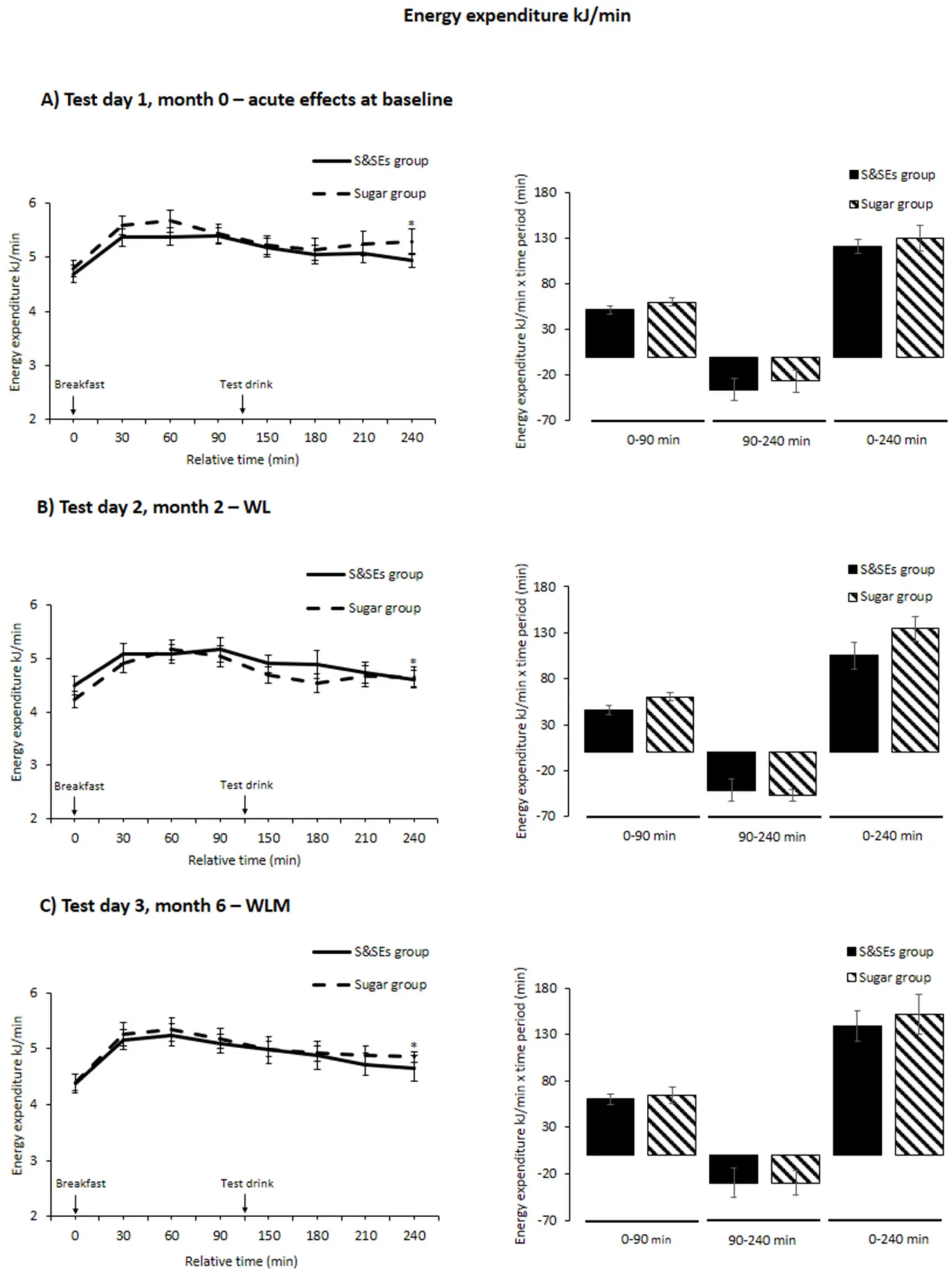
Energy expenditure shown as unadjusted means ± SEM at different time points (**left**) and netAUC (**right**) for all 3 test days: (**A**) test day 1, month 0—acute effects at baseline (S&SEs group, n = 14; Sugar group, n = 11, data from 1 participant were lost due to technical issues, (**B**) test day 2, month 2—WL (S&SEs group, n = 13; Sugar group, n = 9), and (**C**) test day 3, month 6—WLM (S&SEs group, n = 9; Sugar group, n = 7). ANCOVA models were used to test for differences between groups. The models all included participant number, test day, and hood machine as random effects, and age, sex, smoking status, BMI at baseline, and weight change from baseline as fixed effects. Models for repeated measurements also included fasting energy expenditure as a fixed effect. Holm’s method was used for multiple testing adjustments when differences were found. A time–meal interaction was found at time point 240 min across the 3 test days (*p* = 0.048, adjusted means). This difference disappeared (*p* = 0.31) after rerunning the analyses excluding a participant whose data on test day 1 had a very deviating pattern compared to all other participants and the participant’s own data on test days 2 and 3. No differences were found between groups for netAUC (all *p* > 0.05, adjusted means). Abbreviations: netAUC, net area under the curve; SEM, standard error mean; S&SEs, sweeteners and sweetness enhancers; WL, weight loss; WLM, weight loss maintenance. * *p* < 0.05.

No time–meal–test day interaction was found for energy expenditure across the three test days (*p* = 0.95), but there was a time–meal interaction (*p* = 0.006). Post hoc analyses found lower energy expenditure in the S&SEs group compared to the Sugar group at 60 min (0.22 ± 0.10 kJ/min, *p* = 0.02) and 240 min (0.26 ± 0.10 kJ/min, *p* = 0.007). However, after adjusting for multiple testing, only the difference at 240 min remained (*p* = 0.048) (Figure 5, left). After rerunning the analyses excluding a participant whose data on test day 1 had a very deviating pattern compared to the data of all other participants and the participant’s own data on test days 2 and 3, the overall time–meal effect remained (*p* = 0.02) and post hoc analyses still indicated a time–meal effect at 240 min (*p* = 0.04). However, this time–meal effect disappeared after adjusting for multiple testing (*p* = 0.31). The participant was not an outlier, but the participant’s mean curve drew near to the ±3 SD mean curve. Lastly, netAUC energy expenditure did not differ between groups for any of the performed analyses (Figure 5, right; Supplementary Table S2) (all *p* > 0.05).

### 3.6. Glucose and Insulin

**Figure 6:**
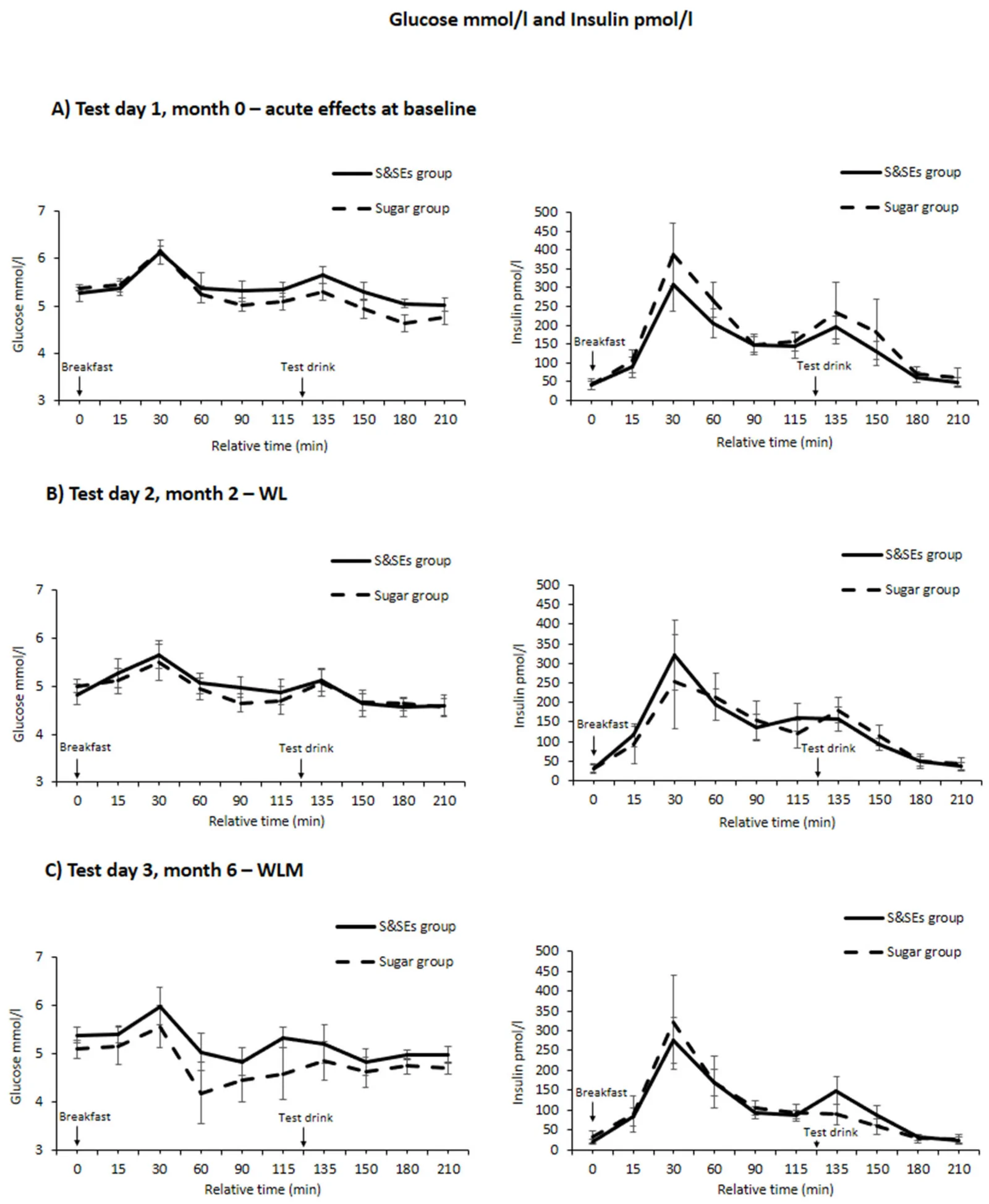
Glucose (**left**) and insulin (**right**) concentrations shown as unadjusted means ± SEM at different time points for all 3 test days: (**A**) test day 1, month 0—acute effects at baseline (S&SEs group, n = 9; Sugar group, n = 6), (**B**) test day 2, month 2—WL (S&SEs group, n = 7, data from 1 participant were lost due to technical issues; Sugar group, n = 6 (glucose), 5 (insulin—data from 1 participant were lost due to technical issues), and (**C**) test day 3, month 6—WLM (S&SEs group, n = 6; Sugar group, n = 3, data from one participant were lost due to technical issues). ANCOVA models were used to test for differences between intervention groups. The models all included participant number and test day as random effects, and test day, age, sex, smoking status, BMI at baseline, and weight change from baseline as fixed effects. Models for repeated measurements also included fasting glucose or insulin as a fixed effect. Holm’s method was used for multiple testing adjustments when differences were found. No differences were found between groups after adjusting for multiple testing (all *p* > 0.05, adjusted means). Abbreviations: SEM, standard error mean; S&SEs, sweeteners and sweetness enhancers; WL, weight loss; WLM, weight loss maintenance.

For glucose, no time–meal–test day interaction (*p* = 0.66) or time–meal interaction (*p* = 0.92) was found. A meal effect across the three test days was found at first (*p* = 0.03), but post hoc analyses showed it to be non-significant (*p* = 0.12) (Figure 6 (left)). The 0–210 min netAUC glucose concentration was higher for the S&SEs group compared to the Sugar group on test day 1, where the Sugar group was only exposed to the breakfast meal and water test drink (79 ± 35 mmol/L × 210 min, *p* = 0.04); however, when adjusting for multiple testing, this difference disappeared (*p* = 0.33). No difference was found between groups for any of the remaining analyses (all *p* > 0.05) (Supplementary Figure S1, left; Table S2). Furthermore, there were no differences in insulin for any of the performed analyses (all *p* > 0.05) (Supplementary Figure S1, right; Table S2).

## 4. Discussion

### 4.1. Summary of Findings

No significant differences were found in fat oxidation between the two groups for any of the performed analyses. Similarly, no significant differences were found for carbohydrate oxidation, energy expenditure, glucose, or insulin. Due to the limited number of participants, it is possible that this was due to type II errors. However, if type II errors do not explain the lack of difference, our findings may suggest that Ace-K/Cyc, when served in a test drink 120 min after a standardized breakfast, has similar effects to water on all these outcomes acutely, after WL, and after 4-month exposure to S&SEs in the diet. Furthermore, the results would indicate that regular consumption of S&SEs for 4 months does not significantly affect the response to a standardized meal compared to the exclusion of S&SEs in the diet.

### 4.2. Fat Oxidation

The lack of significant difference in fat oxidation is in agreement with a similar study by Pearson et al. [33]. In this isoenergetic acute study, no difference in 3 h AUC fat oxidation was found between a test drink with aspartame and water when served with a standardized lunch. However, a time–meal effect at 10 min was found, with fat oxidation being lower after aspartame compared to water [33]. Due to dissimilarities in study design between the current study and the study by Pearson et al. [33], this time–meal effect can neither be confirmed nor refuted. In the current study, the test drinks were served 2 h after the standardized breakfast, thus testing the effect of Ace-K/Cyc alone. Pearson et al. [33], however, tested the effect of aspartame together with other nutrients. Thus, the observed time–meal effect could suggest that aspartame, in combination with other nutrients, has an influence on energy metabolism extending beyond the perception of sweetness. However, due to the limited sample size in the study by Pearson et al. [33], this may be considered exploratory and requires further investigation. Type of NCS and method of administration should always be considered when discrepancies between studies are observed. Pearson et al.’s [33] is, to the best of our knowledge, the only study besides ours that included water as a comparator. In two other isoenergetic acute studies, the non-caloric sweetener sucralose was incorporated into a meal [34,35]. In the study of Mourão et al. [35], there was no difference in 4 h fat oxidation when comparing a meal with sucralose to a meal with sucrose, again supporting our findings. However, in the study of Chern et al. [34], a higher 1.5 h AUC fat oxidation was found after a jelly with sucralose and maltodextrin (a non-sweet carbohydrate) compared to after a jelly with sucrose. While this difference could potentially be attributed to the activation of sweet taste receptors by two different sweeteners (sucralose and sucrose), another plausible explanation for the difference may be the likely different metabolic effects of maltodextrin and sucrose on the sweet taste receptors in the oral cavity and intestine [25]. This argument is supported by the fact that the two otherwise comparable studies including sucralose yielded conflicting results.

Lastly, while no isoenergetic studies have, to the best of our knowledge, evaluated the longer-term effects of NCS on substrate oxidation, one non-isoenergetic study was identified [36]. That study found higher fat and lower carbohydrate oxidation after including a mix of NCS (including Ace-K and Cyc) in the diet for 10 weeks compared to sugar [36]. This finding can likely be attributed to the higher carbohydrate content in the Sugar group and not to an effect of the NCS per se [25]. Thus, this study indirectly aligns with our finding that there were no effects on substrate oxidation after regular exposure to S&SEs for 4 months.

### 4.3. Carbohydrate Oxidation

The similar effects of Ace-K/Cyc and water on carbohydrate oxidation found in this study again align with the results of Pearson et al. [33]. However, in the study of Mourão et al. [35], higher carbohydrate oxidation was found after the meal with sucralose compared to sucrose [35], and Chern et al. [34] found lower carbohydrate oxidation after jelly with sucralose and maltodextrin compared to sucrose [34]. For both studies, issues with standardization might explain the findings, as already pointed out for Chern et al. [34] above. In the study of Mourão et al. [35], the meal with sucralose had a more complex carbohydrate profile, a larger volume, and seemingly a larger protein content, all of which could have affected the results.

### 4.4. Energy Expenditure

The energy and protein content of a meal are the primary factors influencing postprandial energy expenditure [37]. Subsequently, it is expected that isoenergetic drinks/meals lead to the same postprandial energy expenditure if matched for protein content. In the current study, this expected pattern was seen in the netAUC for energy expenditure for all three time periods, indicating that Ace-K/Cyc affects energy expenditure similarly to water and that regular exposure to S&SEs for 4 months did not affect the results.

Contrary to what was seen for substrate oxidation, the results of the current study differ from those of Pearson et al. [33]. In their study, a higher AUC for energy expenditure was found after aspartame compared to water. This result may seem counterintuitive, as there were no differences between the two groups in terms of the total amount of fat and carbohydrate oxidized during the postprandial phase [33]. This could indicate that a type 1 error occurred, which could be explained by the low statistical power. Supporting our results and contrasting the results by Pearson et al. [33] is the study by Chern et al. [34] and an isoenergetic acute study by Prat-Larquemin et al. [38]. In the latter study, no difference in the netAUC for energy expenditure was found between a cheese with aspartame and maltodextrin, a cheese with maltodextrin alone, and a cheese with sucrose [38].

### 4.5. Glucose and Insulin

The lack of significant differences in postprandial glucose and insulin concentrations supports the lack of effect on substrate oxidation and energy expenditure observed in the current study. Furthermore, recent systematic reviews support that NCS and water have similar effects on postprandial glucose and insulin concentrations both acutely and in the long-term [20,21]. The low number of participants in our study does, however, limit our findings.

### 4.6. Strengths and Limitations

To the best of our knowledge, this is the first study to examine the effects of Ace-K/Cyc compared to water on substrate oxidation and energy expenditure acutely and after longer-term exposure to S&SEs in the diet. Furthermore, compared to the isoenergetic studies discussed above, the current study is uniquely set to test the proposed effects on substrate oxidation and energy expenditure as we (1) included water as the comparator, (2) included a standardized breakfast, (3) performed the test days before and after the participants included or excluded S&SEs in their diet, and (4) divided the 4 h netAOC/AUC into three time periods, making it possible to distinguish between the effects of the standardized breakfast and the test drink separately. However, a key limitation of this study is its low statistical power, which is also evident from the large SEMs across the different outcomes. Specifically, the findings related to glucose and insulin should be regarded as exploratory.

## 5. Conclusions

There were no statistically significant differences between Ace-K/Cyc and water in postprandial fat oxidation, carbohydrate oxidation, energy expenditure, or glucose and insulin concentrations acutely (month 0), after a 2-month WL period, or after a 4-month WLM period including or excluding S&SEs in the diet. Furthermore, regular consumption of S&SEs did not significantly affect the responses to a standardized meal compared to excluding S&SEs from the diet. The low number of participants should, however, be considered a limitation of these conclusions.

## Figures and Tables

**Table 1:** Nutritional composition of the standardized breakfast and test drinks.

	Standardized Breakfast	Test Drinks	
Served to	All Participants	S&SEs Group	Sugar Group
Product	Arla Cultura^®^ with Oats and Cranberries ^1^	Atwell^®^0-Calories^®^ with Ace-K/Cyc	-
**Amount**	340 g	5 g	
**Energy**	1476 kJ	0 kJ	
**Fat**	8.8 g	0 g	
**Saturated fat**	3.7 g	0 g	
**Carbohydrates**	40.8 g	0 g	
**Sugar**	24.8 g	0 g	
**Fiber**	13.6 g	0 g	
**Protein**	18.0 g	0 g	
**Salt**	0.34 g	0 g	
**Water**	250 g	400 g	405 g

^1^ Nutritional content of products (Arla Cultura^®^ with oats and cranberries and Atwell^®^), as written on the packaging.

**Table 2:** Baseline characteristics of the 26 participants that completed test day 1.

	S&SEs (n = 15)	Sugar (n = 11)	Total (n = 26)	*p*-Value
**Sex** **Female (n (%)** **Male (n (%)**				
	11 (73)	8 (73)	19 (73)	0.97
	4 (27)	3 (27)	7 (27)	
**Age (y)**	48 ± 11	52 ± 8	49 ± 10	0.27
**Height (cm)**	172.3 ± 7.3	167.4 ± 8.0	170.2 ± 7.9	0.12
**Weight (kg)**	97.6 ± 14.1	89.7 ± 11.4	94.2 ± 13.4	0.13
**BMI (kg/m^2^)**	33.0 ± 5.0	32.0 ± 3.4	32.6 ± 4.3	0.56
**FM (kg)**	40.7 ± 12.2	35.2 ± 7.63	38.3 ± 10.7	0.17
**FFM (kg)**	56.3 ± 8.58	53.5 ± 7.59	55.1 ± 8.14	0.38
**Smoking** **Non-smoker (n (%)**				
	12 (80)	9 (82)	21 (81)	
**Occasional smoker (n (%)** **Daily smoker (n (%)**	1 (7)	1 (9)	2 (8)	
	2 (13)	1 (9)	3 (11)	0.93

Age, height, and smoking status measured at screening in the main study. Fasting body weight measured on test day 1 in the sub-study. Numerical data are shown as means ± SD. *t*-tests were used to test for differences between intervention groups for all numerical outcomes, while categorical data were tested via logistic regression. No differences in baseline characteristics were found (all *p* > 0.05). Abbreviations: FFM, fat-free mass; FM, fat mass; BMI, body mass index; SD, standard deviation; S&SEs, sweeteners and sweetness enhancers; y, year.

## Data Availability

Data are available from the authors upon reasonable request.

## Supplementary Materials

The following supporting information can be downloaded at https://www.mdpi.com/article/10.3390/nu18172863/s1, Table S1: Fasting measurements of fat oxidation, carbohydrate oxidation, energy expenditure, glucose, and insulin on the 3 test days. Table S2: NetAOC/netAUC fat oxidation, carbohydrate oxidation, energy expenditure, glucose, and insulin on the 3 test days. Figure S1: Glucose and insulin concentrations shown as unadjusted means ± SEM for netAUC on all 3 test days.
