# Supplementary material for: Acute and Prolonged Effects of Sweeteners and Sweetness Enhancers on Postprandial Substrate Oxidation, Energy Expenditure, Glucose, and Insulin in Humans—A SWEET Sub-Study
Source: Nutrients. 2026 Sep 2;18(17):2863. doi: 10.3390/nu18172863 (PMC13567529; doi:10.3390/nu18172863)
Supplement: Supplementary file 1 [file nutrients-18-02863-s001.zip › nutrients-4464787-supplementary.pdf]

Supplementary material

***Supplementary Table S1**: Unadjusted and adjusted means±SEM for fasting measurements of fat oxidation, carbohydrate oxidation, energy expenditure, glucose and insulin on the 3 test days.*

	**S&SEs group Unadjusted**	**Sugar group Unadjusted**	**S&SEs group Adjusted[1]**	**Sugar group Adjusted[1]**	**Between-group differences [95% CI] Adjusted[1]**	**P-values (for adjusted means)**
**Fasting fat oxidation (kJ/min)**						
**Test day 1 – month 0 (baseline)**	2.4 ± 0.3	2.3 ± 0.2	3.0 ± 0.4	2.8 ± 0.4	0.1 [-0.5:0.8]	0.68
**Test day 2 – month 2 (after WL)**	2.5 ± 0.3	2.1 ± 0.3	2.7 ± 0.4	2.4 ± 0.4	0.4 [-0.3:1.1]	0.31
**Test day 3 – month 6 (after WLM)**	2.6 ± 0.2	2.7 ± 0.2	2.9 ± 0.4	2.9 ± 0.4	0.0 [-0.9:0.9]	0.99
**Fasting carbohydrate oxidation (kJ/min)**						
**Test day 1 – month 0 (baseline)**	1.1 ± 0.2	1.3 ± 0.2	0.7 ± 0.4	1.0 ± 0.4	-0.3 [-1.0:0.4]	0.37
**Test day 2 – month 2 (after WL)**	0.8 ± 0.3	0.9 ± 0.4	0.8 ± 0.4	1.1 ± 0.4	-0.3 [-1.1:0.5]	0.41
**Test day 3 – month 6 (after WLM)**	0.5 ± 0.2	0.5 ± 0.2	0.5 ± 0.4	0.7 ± 0.4	-0.2 [-1.1:0.8]	0.70
**Fasting energy expenditure (REE) (kJ/min)**						
**Test day 1 – month 0 (baseline)**	4.7 ± 0.2	4.8 ± 0.2	4.8 ± 0.2	5.0 ± 0.2	-0.2 [-0.5:0.1]	0.22
**Test day 2 – month 2 (after WL)**	4.5 ± 0.2	4.2 ± 0.2	4.8 ± 0.2	4.7 ± 0.2	0.0 [-0.3:0.4]	0.82
**Test day 3 – month 6 (after WLM)**	4.4 ± 0.2	4.4 ± 0.2	4.7 ± 0.2	4.8 ± 0.2	-0.1 [-0.5:0.3]	0.66
**Fasting glucose (mmol/l)**						
**Test day 1 – month 0 (baseline)**	5.3 ± 0.1	5.4 ± 0.0	5.2 ± 0.3	5.3 ± 0.3	-0.1 [-0.6:0.4]	0.78
**Test day 2 – month 2 (after WL)**	4.8 ± 0.1	5.0 ± 0.1	4.8 ± 0.3	5.0 ± 0.3	-0.2 [-0.7:0.4]	0.47
**Test day 3 – month 6 (after WLM)**	5.4 ± 0.1	5.1 ± 0.2	5.5 ± 0.3	5.1 ± 0.4	0.5 [-0.2:1.1]	0.18
**Fasting insulin (pmol/l/)**						
**Test day 1 – month 0 (baseline)**	40.3 ± 3.8	42.6 ± 15.3	23.4 ± 18.2	26.5 ± 22.3	-3.1 [-33.4:27.3]	0.84
**Test day 2 – month 2 (after WL)**	32.1 ± 3.6	30.9 ± 11.4	12.6 ± 18.9	12.4 ± 20.9	0.3 [-32.1:32.7]	0.99
**Test day 3 – month 6 (after WLM)**	21.2 ± 1.7	31.5 ± 17.1	5.57 ± 19.5	31.3 ± 22.5	-25.7 [-64.2:12.8]	0.18

Data are shown as unadjusted/adjusted means±SEM. [1]Means were adjusted for participant number, test day, hood machine (when relevant), age at baseline, sex, smoking status, BMI at baseline, and weight change from baseline. ANCOVA models were used to test for differences between groups. No difference between groups was found for any of the outcomes on any of the 3 test days. Abbreviations: REE, resting energy expenditure; S&SEs, **sweeteners and sweetness enhancers**; SEM, standard error mean**;** WL, weight loss; WLM, weight loss maintenance.

Supplementary Table S2 - *Unadjusted and adjusted means±SEM for netAOC/netAUC fat oxidation, carbohydrate oxidation, energy expenditure, glucose and insulin at the 3 test days.*

	S&SEs group Unadjusted	Sugar group Unadjusted	S&SEs group Adjusted[2]	Sugar group Adjusted[2]	Between-group differences [95% CI] Adjusted[1]	P-values (for adjusted means)
**Fat oxidation netAOC (kJ/min × time period)[1]**						
**Test day 1 – month 0 (baseline)**						
**0-90 min**	-30 ± 14	-19 ± 18	-49 ± 36	-31 ± 39	18 [-58:95]	0.63
**90-240 min**	32 ± 26	-6 ± 25	13 ± 36	-18 ± 39	-31 [-108:45]	0.42
**0-240 min**	-70 ± 39	-36 ± 44	-89 ± 36	-48 ± 39	41 [-35:117]	0.29
**Test day 2 – month 2 (after WL)**						
**0-90 min**	-27 ± 12	-27 ± 11	-49 ± 36	-36 ± 39	13 [-72:99]	0.76
**90-240 min**	73 ± 21	8 ± 20	51 ± 36	-2 ± 39	-53 [-139:32]	0.22
**0-240 min**	-64 ± 40	-33 ± 39	-86 ± 36	-43 ± 39	43 [-43:128]	0.32
**Test day 3 – month 6 (after WLM)**						
**0-90 min**	-40 ± 10	-24 ± 43	-60 ± 40	-32 ± 41	28 [-74:130]	0.59
**90-240 min**	-8 ± 21	-11 ± 23	-28 ± 40	-18 ± 41	10 [-92:112]	0.84
**0-240 min**	-85 ± 35	-38 ± 36	-104 ± 40	-45 ± 41	59 [-43:161]	0.26
**Carbohydrate oxidation netAUC (kJ/min × time period)**						
**Test day 1 – month 0 (baseline)**						
**0-90 min**	81 ± 15	78 ± 20	109 ± 38	94 ± 42	15 [-67:97]	0.71
**90-240 min**	-68 ± 27	-20 ± 31	-41 ± 38	-5 ± 42	-36 [-118:46]	0.39
**0-240 min**	192 ± 38	167 ± 50	219 ± 38	182 ± 42	37 [-44:119]	0.37
**Test day 2 – month 2 (after WL)**						
**0-90 min**	73 ± 13	87 ± 14	103 ± 38	102 ± 41	0 [-92:93]	0.99
**90-240 min**	-114 ± 27	-54 ± 23	-85 ± 38	-39 ± 41	-45 [-137:47]	0.33
**0-240 min**	169 ± 45	167 ± 43	198 ± 38	183 ± 41	16 [-76:108]	0.74
**Test day 3 – month 6 (after WLM)**						
**0-90 min**	100 ± 8	90 ± 17	126 ± 42	102 ± 43	25 [-85:135]	0.66
**90-240 min**	-22 ± 24	-19 ± 29	4 ± 42	-7 ± 43	11 [-99:121]	0.84
**0-240 min**	224 ± 31	190 ± 28	250 ± 42	202 ± 43	48 [-62:158]	0.40
**Energy expenditure netAUC (kJ/min × time period)**						
**Test day 1 – month 0 (baseline)**						
**0-90 min**	52 ± 4	60 ± 5	58 ± 14	60 ± 15	-2 [-31:28]	0.92
**90-240 min**	-36 ± 12	-26 ± 12	-29 ± 14	-26 ± 15	-3 [-32:27]	0.85
**0-240 min**	121 ± 7	130 ± 14	128 ± 14	130 ± 15	-2 [-31:28]	0.90
**Test day 2 – month 2 (after WL)**						
**0-90 min**	46 ± 5	61 ± 5	53 ± 14	64 ± 15	-11 [-43:22]	0.51
**90-240 min**	-41 ± 12	-47 ± 6	-34 ± 14	-44 ± 15	10 [-23:42]	0.56
**0-240 min**	105 ± 15	134 ± 14	113 ± 14	138 ± 15	-25 [-58:7]	0.12
**Test day 3 – month 6 (after WLM)**						
**0-90 min**	60 ± 6	65 ± 9	66 ± 16	67 ± 16	-1 [-39:37]	0.96
**90-240 min**	-30 ± 16	-29 ± 13	-24 ± 16	-28 ± 16	4 [-35:42]	0.85
**0-240 min**	139 ± 17	151 ± 22	145 ± 16	154 ± 16	-9 [-47:29]	0.64
**Glucose netAUC (mmol/l x time period)**						
**Test day 1 – month 0 (baseline)**						
**0-115 min**	25 ± 25	3 ± 14	10 ± 31	-23 ± 38	33 [-37:103]	0.35
**115-210 min**	-8 ± 9	-19 ± 13	-24 ± 31	-45 ± 38	21 [-49:91]	0.54
**0-210 min**	22 ± 42	-42 ± 28	7 ± 31	-68 ± 38	75 [5:145]	0.04, 0.33[3]

	S&SEs group Unadjusted	Sugar group Unadjusted	S&SEs group Adjusted[2]	Sugar group Adjusted[2]	Between-group differences [95% CI] Adjusted[1]	P-values (for adjusted means)
**Test day 2 – month 2 (after WL)**						
**0-115 min**	37 ± 11	2 ± 17	-1 ± 38	-40 ± 44	45 [-28:119]	0.22
**115-210 min**	-15 ± 17	0 ± 19	-54 ± 38	-43 ± 44	-11 [-84:62]	0.76
**0-210 min**	27 ± 23	-23 ± 27	-11 ± 38	-66 ± 44	62 [-11:136]	0.09
**Test day 3 – month 3 (after WLM)**						
**0-115 min**	-13 ± 24	-40 ± 26	-50 ± 41	-75 ± 48	24 [-70:118]	0.61
**115-210 min**	-29 ± 11	12 ± 30	-67 ± 41	-23 ± 48	-45 [-139:49]	0.34
**0-210 min**	-47 ± 36	-77 ± 35	-84 ± 41	-112 ± 48	26 [-68:120]	0.58
**Insulin netAUC (pmol/l x time period)**						
**Test day 1 – month 0 (baseline)**						
**0-115 min**	15964 ± 2868	19793 ± 2723	13263 ± 4748	15357 ± 5810	2695 [-7397:12791]	0.65
**115-210 min**	-3394 ± 2236	-2362 ± 2888	-6095 ± 4748	-6799 ± 5810	704 [-8595:10004]	0.88
**0-210 min**	22581 ± 3649	28263 ± 5724	19880 ± 4748	23826 ± 5810	-3946 [-13245:5353]	0.40
**Test day 2 – month 2 (after WL)**						
**0-115 min**	17146 ± 3751	11475 ± 5697	15422 ± 5007	12725 ± 5619	2697 [-7397:12791]	0.60
**115-210 min**	-6851 ± 1790	-2285 ± 1402	-8574 ± 5007	-5489 ± 5619	-3085 [-13179:7008]	0.54
**0-210 min**	22566 ± 4630	22085 ± 6767	20842 ± 5007	18880 ± 5619	1962 [-8132:12056]	0.70
**Test day 3 – month 6 (after WLM)**						
**0-115 min**	14356 ± 2779	14382 ± 3429	12368 ± 5212	14184 ± 6331	-1817 [-13891:10258]	0.76
**115-210 min**	-1603 ± 1772	-3862 ± 1581	-3591 ± 5212	4060 ± 6331	6047 [-11605:12544]	0.94
**0-210 min**	19153 ± 3702	16442 ± 2952	17165 ± 5212	16244 ± 6331	921 [-11154:12996]	0.88

Data are shown as unadjusted/adjusted means±SEM. [1]To illustrate that netAOC provides information about how much fat oxidation is inhibited the netAOC means have been reversed. [2]Means were adjusted for participant number, test day, hood machine (when relevant), age at baseline, sex, smoking status, BMI at baseline, and weight change from baseline. [3]P-value adjusted for multiple testing. ANCOVA models were used to test for differences between groups. Holm's method was used for multiple testing adjustments when differences were found. No difference between groups was found for any of the outcomes after adjusting for multiple testing. Abbreviations: netAOC, net area over the curve; netAUC, net area under the curve; S&SEs, **sweeteners and sweetness enhancers**; SEM, standard error mean; WL, weight loss; WLM, weight loss maintenance.

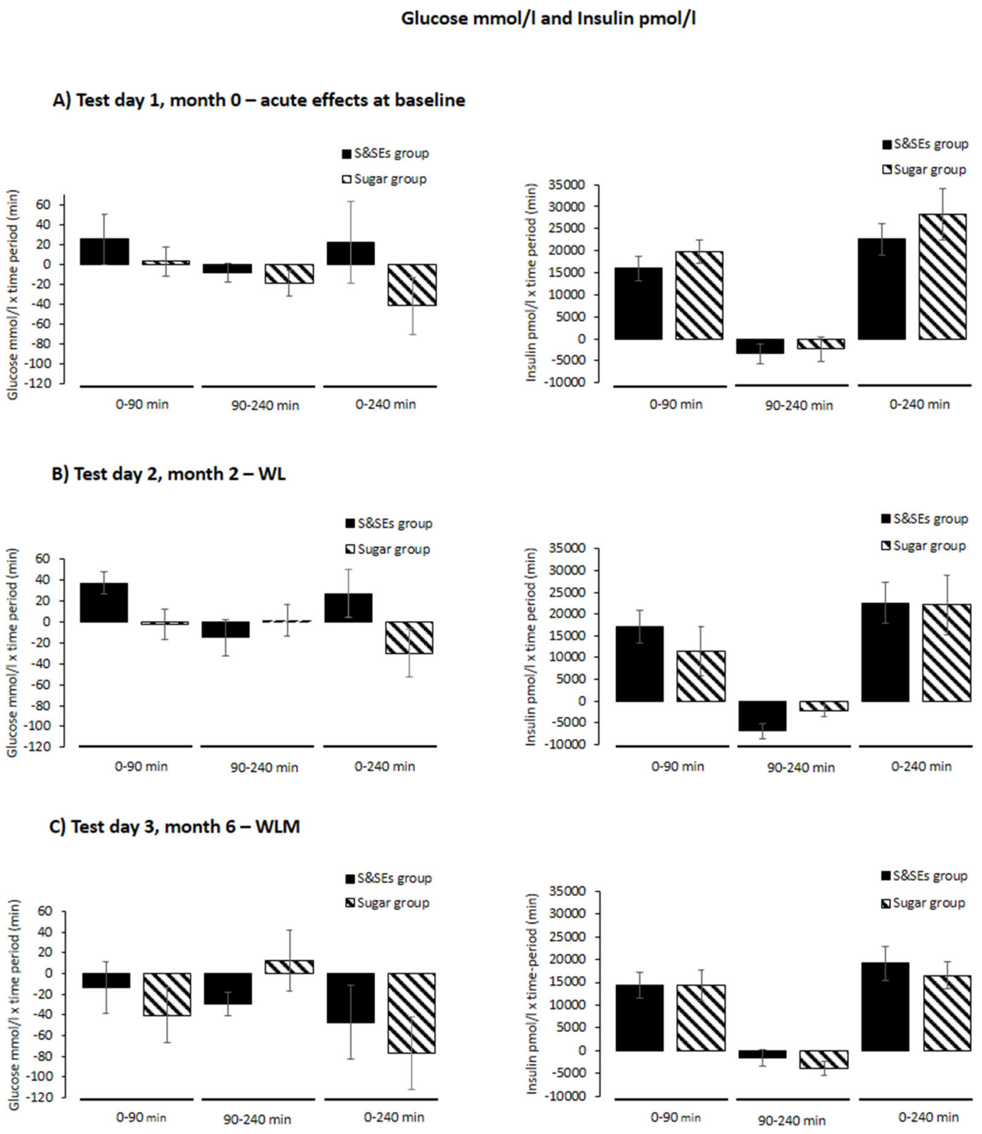

***Supplementary Figure S1*:** Glucose (left) and insulin (right) concentrations shown as unadjusted means±SEM for netAUC on all 3 test days: A) Test day 1, month 0 – acute effects at baseline (S&SEs group, n = 9; Sugar group, n = 6), B) Test day 2, month 2 – WL (S&SEs group, n = 7 (data from one participant were lost due to technical issues); Sugar group, n = 6 (glucose), 5 (insulin – data from one participant were lost due to technical issues) and C) Test day 3, month 6 – WLM (S&SEs group, n = 6; Sugar group, n = 3 (data from one participant were lost due to technical issues). ANCOVA models were used to test for differences between intervention groups. The models all included participant number and test day as random effects, and age, sex, smoking status, BMI at baseline, and weight change from baseline as fixed effects. Models for repeated measurements also included fasting glucose or insulin as a fixed effect. Holm's method was used for multiple testing adjustments when differences were found. No differences were found between groups after adjusting for multiple testing (all p>0.05, adjusted means). Abbreviations: netAUC, net area under the curve; SEM, standard error mean; S&SEs, **sweeteners and sweetness enhancers**; WL, weight loss; WLM, weight loss maintenance.
